# PCSK9: an emerging player in cardiometabolic aging and its potential as a therapeutic target and biomarker

**DOI:** 10.1007/s11357-023-01003-0

**Published:** 2023-12-18

**Authors:** Anna Csiszar, Stefano Tarantini, Andriy Yabluchanskiy, Zoltan Ungvari

**Affiliations:** 1 Vascular Cognitive Impairment, Neurodegeneration, and Healthy Brain Aging Program, Department of Neurosurgery, University of Oklahoma Health Sciences Center, Oklahoma City, OK USA; 2 Oklahoma Center for Geroscience and Healthy Brain Aging, University of Oklahoma Health Sciences Center, Oklahoma City, OK USA; 3 Stephenson Cancer Center, University of Oklahoma Health Sciences Center, Oklahoma City, OK USA; 4 OUHSC-SE International Training Program in Geroscience, Doctoral School of Basic and Translational Medicine, Semmelweis University, Budapest, Hungary

**Keywords:** Aging, Inflammaging, Nonalcoholic fatty liver disease

## Abstract

Proprotein convertase subtilisin/kexin type 9 (PCSK9), renowned for its pivotal role in low-density lipoprotein (LDL) regulation, has emerged as a compelling regulator of cardiometabolic aging. Beyond its well-established involvement in cholesterol metabolism, PCSK9’s multifaceted influence on the aging processes of the cardiovascular and metabolic systems is garnering increasing attention. This review delves into the evolving landscape of PCSK9 in the context of cardiometabolic aging, offering fresh insights into its potential implications. Drawing inspiration from pioneering research conducted by the Pacher laboratory (Arif et al., Geroscience, 2023, PMID: 37726433), we delve into the intricate interplay of PCSK9 within the aging heart and liver, shedding light on its newfound significance. Recent studies underscore PCSK9’s pivotal role in liver aging, suggesting intriguing connections between hepatic aging, lipid metabolism, and cardiovascular health. Additionally, we explore the therapeutic potential of PCSK9 as both a target and a biomarker, within the context of age-related cardiovascular disease.

## Introduction

Proprotein convertase subtilisin/kexin type 9 (PCSK9) is a key enzyme in cholesterol metabolism, known for its role in regulating low-density lipoprotein cholesterol (LDL) levels. However, recent research has unveiled a broader and more intricate role for PCSK9, extending its significance beyond its established cholesterol-related functions. This mini-review delves into the evolving landscape of PCSK9 in the context of cardiometabolic aging, offering fresh perspectives on its potential implications. While PCSK9’s role in cholesterol homeostasis has been extensively studied, its involvement in the aging processes of the cardiovascular and metabolic systems has gained prominence. This emerging understanding, especially in light of recent innovative studies by the Pacher laboratory [1, 2], raises intriguing questions about PCSK9’s multifaceted influence on cardiometabolic aging and its implications for health and disease. Moreover, PCSK9’s dual nature as both a therapeutic target and a potential biomarker holds significant promise. Beyond its therapeutic potential, PCSK9 may offer valuable insights into the aging-related changes occurring in the body, providing a bridge between the molecular mechanisms of aging and liver and cardiovascular health.

## PCSK9 as a therapeutic target

PCSK9 has garnered significant attention as a promising therapeutic target, revolutionizing the landscape of lipid management and cardiovascular disease prevention [3]. Recent advancements in PCSK9 targeting have opened new avenues for intervention and raised the prospect of improved outcomes in individuals at risk of cardiovascular events [3]. Recent years have witnessed a surge in research and drug development efforts focused on PCSK9 inhibition [3]. This heightened interest stems from the pivotal role of PCSK9 in regulating LDL cholesterol levels through its degradation of LDL receptors (LDLRs) in hepatocytes. Inhibition of PCSK9 offers a means to amplify LDLR expression, thereby enhancing the clearance of LDL cholesterol from circulation. These developments are particularly significant in the context of unhealthy cardiometabolic aging, where elevated LDL cholesterol levels are a well-established risk factor for the development of atherosclerosis and cardiovascular disease.

The efficacy of PCSK9 inhibitor monoclonal antibodies in reducing LDL cholesterol levels has been compellingly demonstrated in an extensive array of clinical trials [3]. These biologics effectively disrupt PCSK9-mediated degradation of LDLRs, leading to a substantial reduction in circulating LDL cholesterol. The clinical impact of PCSK9 inhibitor monoclonal antibodies extends beyond LDL cholesterol reduction. Several large-scale trials have not only affirmed their potent lipid-lowering capabilities but have also illuminated their potential to decrease cardiovascular mortality and morbidity. These findings underscore the multifaceted benefits of PCSK9 inhibition and its relevance in the context of cardiometabolic aging.

A recent milestone in PCSK9-targeted therapy involves the inhibition of liver PCSK9 synthesis using a small interfering RNA (siRNA) molecule known as Inclisiran [4, 5]. Approved by the U.S. Food and Drug Administration (FDA), Inclisiran offers a unique approach to PCSK9 modulation. Inclisiran’s mechanism of action involves silencing the expression of PCSK9 mRNA in hepatocytes, effectively reducing the production of PCSK9 protein. Clinical trials have demonstrated its ability to lower LDL cholesterol levels to a degree comparable to that achieved with monoclonal antibodies. This innovative approach holds promise for individuals with hypercholesterolemia and contributes to the expanding armamentarium of PCSK9-targeted therapies.

Further diversifying the field of PCSK9 inhibition, the oral PCSK9 inhibitor MK-0616 has entered the realm of clinical development [6]. While the majority of PCSK9 inhibitors have been administered via subcutaneous injections, MK-0616 offers the convenience of an oral route. The development of an oral PCSK9 inhibitor represents a significant step forward, potentially enhancing patient adherence and treatment accessibility. The progression of MK-0616 through clinical trials underscores the ongoing innovation in PCSK9-targeted therapies.

Notably, PCSK9 inhibition has demonstrated its potential to deliver benefits that transcend its primary mechanism of action. While initially celebrated for its remarkable capacity to lower LDL cholesterol levels, PCSK9 inhibition has exhibited broader implications for cardiovascular health. Clinical trials have highlighted the potential of PCSK9 inhibitors to reduce cardiovascular mortality and morbidity, suggesting that their impact extends to multiple facets of cardiometabolic aging [3]. The emerging evidence underscores the intricate interplay between PCSK9, lipid metabolism, and cardiovascular health, encouraging continued exploration of its therapeutic potential in the context of aging and age-related cardiovascular diseases.

In conclusion, PCSK9 has emerged as a pivotal therapeutic target with the capacity to redefine the management of hypercholesterolemia and cardiovascular risk. Recent advancements in PCSK9-targeted therapies, including monoclonal antibodies, Inclisiran, and oral inhibitors, offer a spectrum of options for clinicians and patients. Moreover, the potential benefits of PCSK9 inhibition extend beyond LDL cholesterol reduction, emphasizing its relevance in addressing age-related cardiovascular dysfunction. These developments hold promise for the future of cardiometabolic aging management, presenting new opportunities to mitigate the burden of cardiovascular disease in aging populations.

## PCSK9’s role in liver aging

The liver represents a vital hub in cardiometabolic aging, orchestrating a multitude of processes that impact cardiovascular and overall health. During aging, the liver undergoes substantial pathological transformations, which can have profound consequences on systemic physiology [7–15]. Understanding the intricate interplay between liver aging and cardiovascular health is imperative for elucidating the broader implications of PCSK9 in the context of aging.

In a recent intriguing study published in *Geroscience*, Arif et al. harnessed data-driven techniques to unveil PCSK9 as a pivotal regulator in the aging of the liver [2]. Their investigation leveraged an established rat model of aging (Fischer F344 rats) providing valuable insights into the role of PCSK9 in liver aging. Arif et al. meticulously characterized the transcriptomics landscape changes induced by aging in the liver [2]. Through comprehensive analyses, they delineated the shifts in gene expression patterns that occur as the liver ages, which associate with the development of nonalcoholic fatty liver disease (NAFLD). These changes encompassed a range of biological processes and pathways, illuminating the intricate molecular alterations associated with hepatic aging. Their work successfully elucidated the hallmark features of the aging liver, which included mitochondrial dysfunction, heightened oxidative/nitrative stress, impaired NAD+ and amino acid metabolism, cell death (apoptosis and senescence), inflammation, lipid accumulation, and fibrotic remodeling. Furthermore, their findings were validated through examination in both animal and human cohorts. A standout aspect of the study by Arif et al. was the creation and meticulous analysis of a liver aging gene co-expression network. This network-based approach is widely employed in investigating functional connections among various molecular elements in disease-related studies. In this context, it allowed the researchers to explore the relationships and interactions among genes implicated in liver aging. Of particular significance, Arif et al. [2] identified a strong association between PCSK9 and cholesterol and lipid biosynthesis pathways within the context of liver aging. This discovery raised intriguing questions about the role of PCSK9 in lipid metabolism and its potential impact on age-related metabolic dysregulation. Furthermore, analysis of human transcriptomics data by Arif et al. [2] revealed an intensification of aging-related pathways in subjects whose liver expressed high levels of PCSK9. Additionally, the examination of single-cell transcriptomics data from liver tissues confirmed that PCSK9 was predominantly expressed in hepatocytes, and its expression increased with age. Collectively, Arif et al.’s findings provided compelling evidence that PCSK9 is highly expressed in hepatocytes and likely plays a critical role in the aging process of the liver. The comprehensive application of systems and network biology analyses led to the formulation of a hypothesis and the identification of PCSK9 as a significant regulator in liver aging.

The implications of PCSK9’s hepatic expression in aging are far-reaching. Not only does it underscore the multifaceted role of PCSK9 in cardiometabolic aging but it also suggests potential connections between liver aging and cardiovascular dysfunction. Further exploration of these links promises to expand our understanding of the intricate interplay between liver health, metabolic aging, and cardiovascular outcomes.

### PCSK9 inhibition and nonalcoholic fatty liver disease (NAFLD)

The link between PCSK9 inhibition and nonalcoholic fatty liver disease (NAFLD) offers intriguing insights into the broader implications of targeting PCSK9 in the context of cardiometabolic aging [16–23]. Notably, observations from various studies have pointed to the potential of PCSK9 inhibition in attenuating NAFLD [17, 20, 23]. NAFLD, a common manifestation of metabolic dysfunction, is characterized by the accumulation of fat in the liver. Research has indicated that PCSK9 inhibition may have a positive impact on liver health, potentially mitigating the development and progression of NAFLD [17, 20]. The findings align with the growing body of evidence supporting the beneficial effects of PCSK9 inhibition in various models of both alcoholic and NAFLD [21–23]. These consistencies across different studies underscore the potential of PCSK9 modulation as a versatile strategy for addressing fatty liver diseases, which frequently accompany the aging process.

The implications of PCSK9 inhibition in attenuating NAFLD extend beyond liver health. Given the intricate connections between liver function and cardiovascular outcomes, these observations hold relevance in the broader context of age-related cardiometabolic aging. The potential of PCSK9 inhibition to address liver pathology aligns with the comprehensive approach required for managing age-related metabolic disorders and cardiovascular risk.

## Role of PCSK9 in cardiovascular aging

The relationship between PCSK9 and cardiovascular aging is an area of increasing interest, with emerging evidence highlighting the multifaceted impact of PCSK9 on age-related cardiovascular dysfunction.

A significant step in unraveling the connection between PCSK9 and cardiovascular aging involved a highly significant study by the Pacher laboratory that measured plasma PCSK9 levels in individuals across various age groups [1]. This investigation sought to discern patterns of PCSK9 expression in the context of aging, shedding light on its potential role in age-related cardiovascular changes. Their findings revealed a substantial increase in PCSK9 plasma levels among aging subjects. This evidence was further reinforced by a large-scale biological network analysis and database platform, iNetModels, which demonstrated a strong positive correlation between age and PCSK9 levels in participants from the SCAPIS-SciLifeLab longitudinal wellness study [1]. Furthermore, they observed a positive association between serum PCSK9 levels and age-related cardiovascular dysfunction and hypertrophy in human subjects [1]. To comprehensively validate these associations, the researchers conducted a thorough hemodynamic analysis, employing techniques such as echocardiography and a pressure-volume approach in a translational aging rat model. The results confirmed comparable associations of serum PCSK9 levels and cardiac dysfunction in aging rats. Aging hearts, similarly to aging livers [2], exhibited all the characteristic hallmarks of the aging [1]. Exploring the connection between PCSK9 and age-related cardiovascular dysfunction has led to the recognition of a complex web of interactions. Notably, this study has unveiled a link between liver inflammation, triglyceride accumulation, and cardiac dysfunction in the context of aging [1], extending the findings of preclinical [24] and clinical studies [25]. Understanding these interrelationships is crucial for understanding how PCSK9 fits into the broader cardiovascular aging landscape.

A significant breakthrough in this field has been the investigation of PCSK9 inhibition as a potential strategy to improve age-related cardiovascular dysfunction. Chronic treatment of aging rats with a PCSK9 inhibitor antibody has been shown to restore LDL receptor levels in the liver, leading to a reduction in serum LDL and oxidized LDL (ox-LDL) levels [1]. Moreover, this treatment effectively mitigated age-related pathological changes observed in the heart, including issues such as mitochondrial dysfunction, oxidative stress, inflammation, and cell death [1]. These findings suggest that PCSK9 inhibition offers promise not only in ameliorating lipid-related cardiovascular risk but also in addressing broader age-related cardiac pathologies. Interestingly, PCSK9 inhibition not only improved the age-related cardiovascular dysfunction and pathology but it also attenuated the age-associated development of NAFLD. This finding is consistent with the observed beneficial effects of PCSK9 inhibition in various models of both alcoholic and NAFLD [21–23].

Surprisingly, an examination of PCSK9 levels in mouse, rat, or human hearts, utilizing multiple techniques such as transcriptomics, single-cell and spatial transcriptomics, RT-PCR, and ELISA, revealed negligible or near-absent expression, if any [1]. This is in stark contrast to results obtained in the liver, which abundantly express PCSK9, with levels increasing further as individuals age [1]. This indicates that the liver serves as the primary source of circulating PCSK9, aligning with the comparable effectiveness of liver-specific PCSK9 inhibition in lowering LDL levels in both in preclinical and clinical trials [4, 5].

Dr. Pacher’s team proposed a compelling hypothesis, suggesting that age-related processes, including inflammation, fibrosis, oxidative stress, and fat accumulation, trigger an increase in PCSK9 production [1]. This upregulation, in turn, leads to a reduction in the number of LDL receptors (LDLRs) in the liver, resulting in the release of LDL, oxidized LDL (ox-LDL), and PCSK9 into circulation, contributing to both local and systemic effects. These effects may play a role in the progression of age-related cardiovascular changes, encompassing mitochondrial dysfunction, oxidative stress, inflammation (TNF-α), myocardial hypertrophy, and fibrotic remodeling [1]. Collectively, these changes ultimately result in the manifestation of cardiovascular dysfunction. Notably, the inhibition of PCSK9 appears to offer a promising avenue for ameliorating age-related cardiac and hepatic pathologies by targeting both systemic and local factors.

While it is plausible to attribute the positive effects of PCSK9 inhibition on the heart to indirect mechanisms, such as the reduction in oxidative stress, inflammation, and circulating cholesterol, there is also the possibility of direct impacts of PCSK9 on both the heart and liver. These direct effects warrant further exploration in future studies, as they could provide valuable insights into the intricate relationships between PCSK9, cardiovascular aging, and age-related pathologies.

## Future directions and perspectives

The evolving landscape of PCSK9 in cardiometabolic aging opens avenues for future research and underscores its far-reaching implications for both understanding the aging process and developing innovative interventions. To comprehensively unravel PCSK9’s role in cardiometabolic aging, further research is needed to investigate the regulation and synthesis of PCSK9 in the aging liver. A deeper understanding of the molecular mechanisms governing PCSK9 production in hepatocytes, especially in the context of aging, can provide crucial insights into its impact on lipid metabolism and age-related liver pathologies. Understanding the role of lifestyle factors, including alcohol consumption, is especially important in this regard. While indirect effects of PCSK9 inhibition on the heart and liver have been well-documented, exploring potential direct impacts of PCSK9 on both organs is an exciting frontier. Investigating the molecular interactions and signaling pathways through which PCSK9 may exert direct effects can elucidate its role in age-related cardiovascular and hepatic changes. Preclinical and clinical studies should explore how PCSK9 modulation, through established and emerging therapeutic strategies, can be tailored to address age-related cardiovascular dysfunction and metabolic derangements. Personalized approaches based on an individual’s PCSK9 profile may hold promise in optimizing interventions. PCSK9’s potential as a biomarker for age-related cardiovascular disease also warrants further investigation. Clinical studies and population-based research can help establish PCSK9’s utility in predicting, diagnosing, and monitoring age-related cardiovascular conditions. A deeper understanding of PCSK9’s biomarker potential can enhance risk assessment and guide early interventions in aging populations.

## Conclusions

In conclusion, PCSK9’s association with cardiovascular aging is a rapidly evolving area of research with significant implications for our understanding of age-related cardiovascular disease. Studies measuring plasma PCSK9 levels, along with investigations into the links between liver health, lipid metabolism, and cardiac function, have begun to uncover the intricate relationships between PCSK9 and cardiovascular aging. The cumulative evidence from various studies suggests that PCSK9 inhibition offers a promising avenue for ameliorating age-related cardiac and hepatic pathologies by targeting both systemic and local factors. Importantly, a recent multivariate genome-wide analysis of aging-related traits identified PCSK9 as a potential new drug target for healthy aging [26]. As the understanding of PCSK9’s multifaceted role in aging and age-related diseases continues to evolve, exploring the therapeutic potential of PCSK9 inhibition in the context of cardiometabolic aging remains an exciting and dynamic area of research.
